# Educational inequalities in self-rated health and emotional exhaustion among workers during the COVID-19 pandemic: a longitudinal study

**DOI:** 10.1007/s00420-022-01931-y

**Published:** 2022-11-02

**Authors:** G. Hulsegge, I. Eekhout, H. A. van de Ven, A. Burdorf, K. M. Oude Hengel

**Affiliations:** 1 Unit Healthy Living, Netherlands Organization for Applied Scientific Research TNO, Sylviusweg 71, 2333 BE Leiden, The Netherlands; 2 Department of Public Health, Erasmus University Medical Center, P.O. Box 2040, 3000 CA Rotterdam, The Netherlands

**Keywords:** Educational inequalities, Emotional exhaustion, General health, Pandemic

## Abstract

**Objective:**

This study aimed to investigate trends in educational inequalities in poor health and emotional exhaustion during the pandemic among workers, and differences in trends between men and women.

**Methods:**

Five waves (2019–2021) from the longitudinal study ‘the Netherlands Working Conditions Survey COVID-19 study’ were used (response rates: 32–38%). Generalized logistic mixed models were used to estimate the changes in absolute and relative educational inequalities in poor health and emotional exhaustion for all workers (*n* = 12,479) and for men and women, separately.

**Results:**

Low and intermediate educated workers reported more often poor health (OR 2.54; 95% CI 1.71–3.77 and OR 2.09; 95% CI 1.68–2.61, respectively) than high educated workers. Intermediate educated women (OR 0.49; 95% CI 0.37–0.64) reported less emotional exhaustion than high educated women, but no differences were observed among men. The prevalence of poor health first decreased across all educational levels until March 2021, and bounced back in November 2021. A similar pattern was found for emotional exhaustion, but for low and intermediate educated workers only. Relative educational inequalities in poor health reduced among men during the pandemic, and absolute differences decreased among men and women by 2.4–2.6%. Relative educational inequalities in emotional exhaustion widened among men only. Absolute differences in emotional exhaustion first increased among both men and women, but narrowed between the last two waves.

**Discussion:**

Socioeconomic inequalities for poor self-rated health remained but narrowed in relative and absolute terms during the pandemic. With regard to emotional exhaustion, socioeconomic inequalities returned to pre-COVID-19 levels at the end of 2021.

**Supplementary Information:**

The online version contains supplementary material available at 10.1007/s00420-022-01931-y.

## Introduction

The COVID-19 pandemic has created immense challenges for the society and health care system. Even though all countries have been heavily affected by the pandemic, the infection and mortality rates due to an infection with COVID-19 are not equally distributed among the population within countries (Abedi et al. 2021; Upshaw et al. 2021; Beese et al. 2022). Due to their worse living conditions, lifestyle behavior, and more comorbidity, people with a lower educational level have a higher risk to be infected with and to die due to COVID-19 compared to people with a higher education level (Hawkins et al. 2020).

From an occupational perspective, not only the direct health consequences of the pandemic are important but also the indirect effects on health, and differences between vulnerable groups of workers (Burdorf et al. 2021). Governments implemented measures to curb the curves of infection rates and accompanying hospitalizations, and have therewith created unprecedented disruptions in social interactions outside the household. Workers who were able to work from home were mainly high educated workers (Eurofound 2020), whereas many essential workers—who are vital for the core function of the society like nurses, bus drivers and day care workers—are mainly low to intermediate educated and had to go to the workplace during the lockdowns. After re-opening parts of the society during the pandemic, workers with a low socioeconomic position—such as hairdressers, waiters and retail workers—returned to their workplace outside the household, whereas high socioeconomic position knowledge workers largely remained working from home (Eurofound 2020).

The question is whether the differential impact of the governmental measures across socioeconomic groups also affected their health differently. Before the COVID-19 pandemic, literature consistently showed large inequalities in health and life expectancy between people with a low education or low income and the more advantaged, due to different exposure to health risks and access to high-quality care (OECD 2016). In 2019, only 63% of European men with a low education were in very good or good health compared to 80% of the highly educated (Eurostat 2021). Also, people with a low socioeconomic position are at a higher risk to develop certain mental health problems—such as depression—compared to those with a high socioeconomic position (WHO 2014). An exception is emotional exhaustion. The prevalence of emotional exhaustion is higher among people with a high educated level compared to those with a low education level in the past ten years in the Netherlands (Houtman et al. 2021).

During the pandemic, many cross-sectional studies have been conducted on the effects of the pandemic on health, and in particular on burn-out among health care workers (Cenat et al. 2021; Clemente-Suárez et al. 2021). Research on previous pandemics and other shocks (e.g., economic recession) showed an indirect and negative impact on socioeconomic inequalities in health or income (Backhaus et al. 2022). It can be hypothesized that negative trends in socioeconomic inequalities might also occur due to the COVID-19 pandemic (Arthi and Parman 2021). Recent cross-sectional studies already showed socioeconomic inequalities in self-perceived health during the pandemic (Zhang et al. 2022; Kim and Kim 2021), and these inequalities are even larger in countries with higher number of coronavirus deaths (Kim and Kim 2021). Longitudinal studies investigating the effects of the pandemic on the general or mental health of other working populations are scarce. A few longitudinal studies have been conducted in the first months of the pandemic and showed an increase in poor mental health among the general population (Pierce et al. 2020; Eurofound 2021), in particular among female workers (Meyer et al. 2021). However, it is still unknown how the pandemic affected self-perceived and mental health of the general working population after the first few months in the pandemic, and whether the possible effects differed by educational groups. Longitudinal studies with a longer follow-up are needed to gain insight into the trends in socioeconomic inequalities, to be able to decide if additional policies or interventions are needed to mitigate the potential long-term effects of the pandemic on health inequalities (Khalatbari-Soltani et al. 2020). The current study will contribute to the knowledge on socioeconomic health inequalities in several ways. This study is embedded in a longitudinal cohort study among the Dutch working population, with one measurement before and four follow-up measurements during the pandemic. Therewith, it is possible to provide insight into health trends over time across socioeconomic groups. Within research on socioeconomic health inequality, Mackenbach and Kunst (1997) discussed the importance to measure inequality from two perspectives, namely the relative risk (i.e., the ratio of rates across socioeconomic groups) and absolute differences across educational groups (i.e., the difference in rates or means between socioeconomic groups). These two different measures have different pros and cons, and a fall or increase in overall level mental or general health might lead to differences in the magnitude or even conclusions about trends in socioeconomic health inequalities (Moser et al. 2007; van Zon et al. 2015). Therefore, both relative risks and absolute differences across educational groups will be estimated in the current study.

The objectives of the study were therefore (1) to investigate how educational inequalities in self-perceived health and emotional exhaustion evolved during the Covid-19 pandemic, and (2) whether these differed between female and male.

## Methods

### Study design and study population

A longitudinal study was conducted with one wave before the pandemic and four during the pandemic. This cohort study, ‘the Netherlands Working Conditions Survey-COVID-19 (NWCS-COVID-19) cohort study’ (Oude Hengel et al. 2021), is an on-going follow-up study of the annual Netherlands Working Conditions Survey (NWCS) of 2019. The study population of the NWCS 2019 was selected by Statistics Netherland and consisted of workers between the age of 15 and 74 years. The study of NWCS has been extensively described elsewhere (Hooftman et al. 2020). For the NWCS-COVID-19 cohort study, a group of participants that granted permission in 2019 were approached again to participate in the current study. Participants received four follow-up online questionnaires on a variety of topics, including sociodemographic factors, working conditions, and health. The TNO Internal Review Board approved the study and assessed the NWCS-COVID-19 cohort study as not being subject to the requirements of the Medical Research (Human Subjects) Act (ID number: 2019-061 for Wave 1; ID number: 2020-057 for Wave 2–4; ID number: 2021-101 for Wave 5). The study followed all recommendations with regard to the privacy aspects including an informed consent, information letter and the possibility to voluntarily discontinue participation in the study.

After baseline (Wave 1; November 2019), the second Wave took place in July 2020 (*n* = 10,115; 38% response), which is just after the peak of the COVID-19 hospitalization in March 2020. Some governmental measures were just relaxed (e.g., primary schools, day care and all sectors were open). Wave 3 took place in November 2020 (*n* = 9475; 36% response) when the number of COVID-19 infections as well as hospitalizations increased, and restaurants, bars and the entertainment industry were closed, and non-essential shops had restricted opening hours. Wave 4 took place in March 2021 (*n* = 8553; 33% response), in which governmental measures were further restricted as non-essential shops were only open upon an appointment and an evening curfew from 9.00 p.m. was introduced. Vaccination was started among health care workers and vulnerable groups. Wave 5 took place in November–December 2021 (*n* = 8099; 32% response), in which governmental measures were restricted (e.g., restaurants were closed, but schools were open) after a relaxation during the summer season. There was a high vaccination rate among the working population but the booster vaccination was not yet introduced.

Of the 15,702 NWCS-COVID-19 participants that participated in at least two waves, 101 participants were excluded because they were younger than 18 years old or older than 66 years, or no information was available on relevant health outcomes at baseline. Of the remaining workers, 2371 participants were excluded because they were not working during their first follow-up measurement. In the last step, 796 workers were excluded because no information was available on either self-rated health or emotional exhaustion at any follow-up measurement. The final analytic sample consisted of 43,081 observations and 12,479 participants.

### Outcomes

#### General health

Self-rated health was measured with a single-item question. Respondents could indicate whether their health was very good, good, fair, poor or very poor. This single-item question on self-rated health is a strong predictor of morbidity and mortality (DeSalvo et al. 2006). Self-rated health was dichotomized into poor health (very poor, poor, or fair) and good health (good or very good).

#### Emotional exhaustion

Emotional exhaustion was measured with five items based on the emotional exhaustion scale from the validated Utrecht Burnout Scale (UBOS) (Schaufeli and van Dierendonck 2000). Using a seven-point scale ranging from never (1) to every day (7), respondents were asked to report the applicability of five statements that refer to emotional exhaustion (e.g., “I feel emotionally exhausted by my work” and “At the end of a working day, I feel empty”). The mean score of the five items was calculated and ranged from 1 to 7. Based on the mean cut-off value of 3.2 (Hooftman et al. 2020), the score was dichotomized into ‘not emotionally exhausted’ and ‘emotionally exhausted’.

### Educational level

Level of education was assessed as the highest level achieved at baseline. Educational level was classified as either low (intermediate secondary education or less), intermediate (higher general secondary education or intermediate vocational education) or high (higher vocational education or university).

### Covariates

Age, sex, household composition and working hours were included as covariates. Working hours and age were included as continuous variables and sex as dichotomous variable. Household composition was categorized as ‘having a partner and children’, ‘having a partner but no children’, and ‘single/other’.

### Statistical analyses

Generalized logistic mixed models were fitted using maximum likelihood estimation for self-rated general health and emotional exhaustion with two levels to account for repeated measurements. Differences across educational strata were estimated using interaction terms between educational position and survey wave. The survey wave was included as categorical variable as we observed a non-linear trend over time in both health and emotional exhaustion. The models were adjusted for gender and age centered at the mean. All analyses were checked for household composition and working hours as potential confounder, but this was not the case and these variables were therefore not included in the final models. The relative differences between the educational groups are expressed as the odds ratio for the interaction between survey wave and educational position. The absolute differences between the groups were estimated by obtaining the predicted values for health and emotional exhaustion at each wave for each participant, based on the models adjusted for age and stratified by gender. All models were estimated with the “glmmTMB” function of the “glmmTMB” package (Brooks et al. 2017) in R statistical software (R Core Team 2021).

## Results

The study population consisted of 12,479 workers, of which a small majority was female (53.2%), the mean age at baseline was 45.7 (SD 11.8) years and the majority was highly educated (58.4%; Table 1). At baseline, almost one fifth of the sutdy population had a poor health (19.9%) or was emotional exhausted (17.6%). Low educated workers were less often female (48.6%) and of a higher age (50.7 years) compared to intermediate and high educated workers. At baseline, the prevalence of poor health was higher among low educated workers (25.7%), while high educated workers reported most often emotional exhaustion (19.0%).

**Table 1:** Characteristics of the study population (*n* = 12,479)

	All	Educational level		
		Low (*n* = 951)	Intermediate (*n* = 4237)	High (*n* = 7291)
Age (mean, SD)	45.7 (11.8)	50.7 (11.3)	46.9 (11.9)	44.4 (11.6)
Gender (% female)	6645 (53.2%)	463 (48.7%)	2121 (50.1%)	4061 (55.7%)
Household composition at baseline				
Having a partner and children	5970 (47.8%)	332 (34.9%)	2035 (48.0%)	3603 (49.4%)
Having a partner but no children	3927 (31.5%)	379 (39.9%)	1305 (30.8%)	2243 (30.8%)
Single or other	2582 (20.7%)	240 (25.2%)	897 (21.2%)	1445 (19.8%)
Health				
General health (% poor)				
Wave 1 (November 2019)	2487 (19.9%)	244 (25.7%)	1000 (23.6%)	1243 (17.0%)
Wave 2 (July 2020)	1365 (16.2%)	136 (21.2%)	525 (18.5%)	704 (14.3%)
Wave 3 (November 2020)	1223 (15.2%)	118 (21.0%)	484 (18.1%)	621 (13.0%)
Wave 4 (March 2021)	1050 (14.8%)	96 (19.1%)	414 (17.2%)	540 (12.8%)
Wave 5 (November 2021)	1231 (19.9%)	107 (24.7%)	443 (21.8%)	681 (18.4%)
Emotional exhaustion (% yes)				
Wave 1 (November 2019)	2201 (17.6%)	149 (15.7%)	665 (15.7%)	1387 (19.0%)
Wave 2 (July 2020)	1403 (17.0%)	96 (15.8%)	414 (15.0%)	893 (18.3%)
Wave 3 (November 2020)	1369 (17.3%)	77 (14.4%)	397 (15.1%)	895 (18.9%)
Wave 4 (March 2021)	1162 (16.7%)	57 (12.0%)	319 (13.6%)	786 (18.9%)
Wave 5 (November 2021)	1148 (18.7%)	69 (16.2%)	340 (16.7%)	739 (20.0%)

**Fig. 1:**
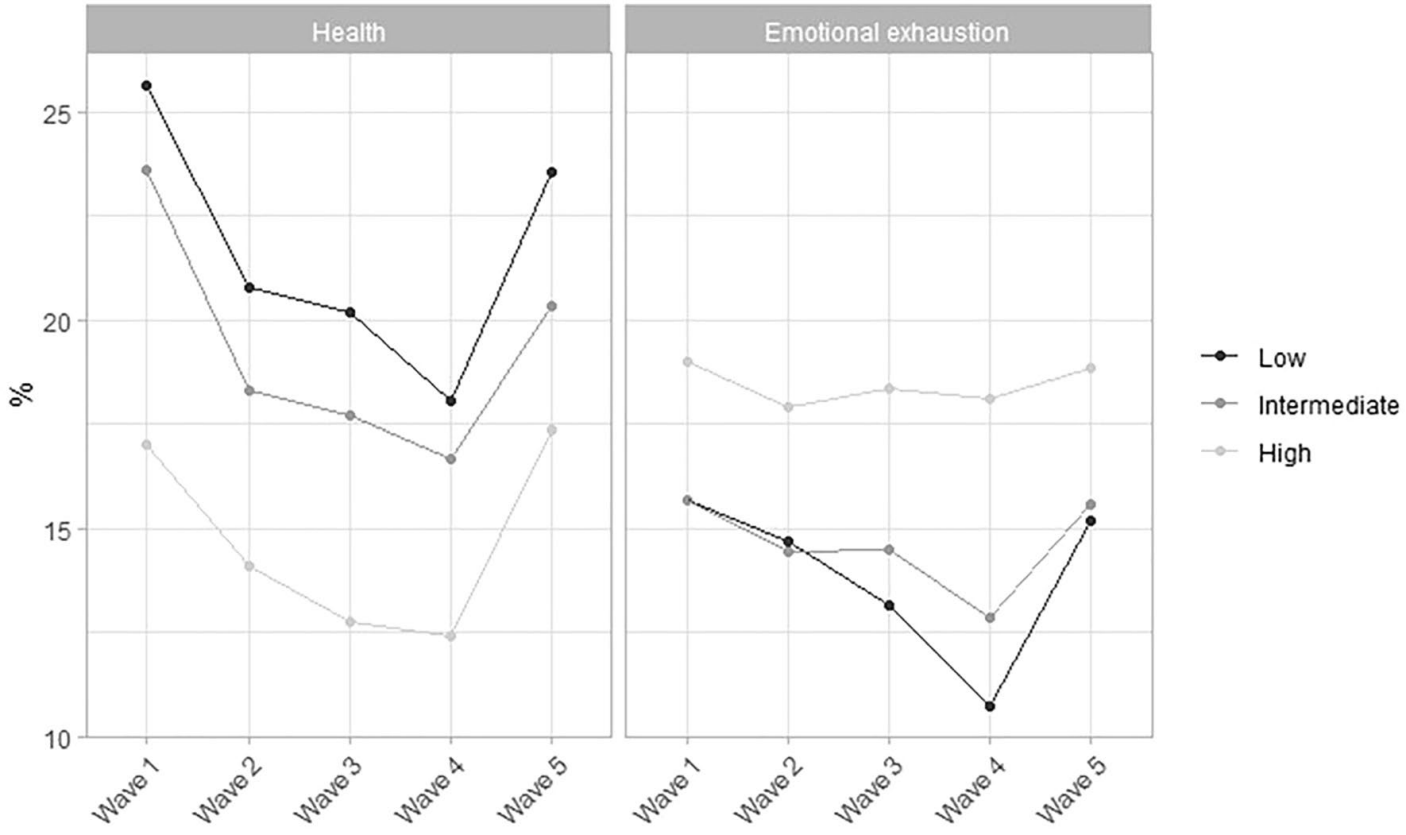
Prevalence of workers with poor health and emotional exhaustion for each educational group and each wave, stratified for men and women

Across all groups during the Covid-19 pandemic, the prevalence of poor health shows a downward trend until March 2021 (Wave 4), but increased again between Wave 4 and 5. While the prevalence of emotional exhaustion showed a similar pattern in low and intermediate educated workers, the prevalence remained relatively high over time in high educated workers (Fig. 1). Supplementary file 1 presents the prevalence of poor health and emotional exhaustion, stratified for men and women.

### Self-rated health

At baseline, low educated workers reported 2.54-fold (95% CI 1.71–3.77) and intermediate educated workers reported 2.09-fold (95% CI 1.68–2.61) higher odds of poor health compared to high educated workers (Table 2). The relative educational inequalities significantly narrowed between intermediate and high educated workers during the pandemic. However, only a statistically significant interaction was found at Wave 5 compared to baseline (OR: 0.67 (95% CI 0.52–0.87)). A similar pattern was found between low and high educated workers at Wave 5, but this was not statistically significant.

**Table 2:** Relative changes over time^a^ in socioeconomic inequality in poor health and emotional exhaustion for the entire study population and stratified by gender

	Poor health			Emotional exhaustion		
	All^b^	Female^c^	Male^c^	All^b^	Female^c^	Male^c^
	OR (95%CI)	OR (95%CI)	OR (95%CI)	OR (95%CI)	OR (95%CI)	OR (95%CI)
Educational level						
Low	**2.54 (1.71–3.77)**	**2.94 (1.68–5.12)**	**2.41 (1.39–4.19)**	0.87 (0.58–1.31)	0.66 (0.10–1.07)	1.23 (0.66–2.29)
Intermediate	**2.09 (1.68–2.61)**	**1.52 (1.14–2.04)**	**2.90 (2.09–4.02)**	**0.71 (0.56–0.90)**	**0.49 (0.37–0.64)**	1.08 (0.75–1.55)
High	Ref	Ref	Ref	Ref	Ref	Ref
Time^d^						
Wave 1 (November 2019)	Ref	Ref	Ref	Ref	Ref	Ref
Wave 2 (July 2020)	**0.59 (0.50–0.69)**	**0.61 (0.50–0.74)**	**0.58 (0.45–0.75)**	0.93 (0.81–1.08)	0.92 (0.78–1.09)	0.97 (0.76–1.25)
Wave 3 (November 2020)	**0.50 (0.43–0.59)**	**0.53 (0.43–0.65)**	**0.48 (0.37–0.62)**	1.05 (0.91–1.22)	1.08 (0.91–1.28)	0.99 (0.77–1.28)
Wave 4 (March 2021)	**0.48 (0.40–0.57)**	**0.41 (0.33–0.52)**	**0.61 (0.47–0.80)**	1.03 (0.88–1.20)	0.92 (0.77–1.11)	1.28 (0.98–1.66)
Wave 5 (November 2021)	**1.20 (1.02–1.41)**	1.20 (0.97–1.47)	1.20 (0.92–1.55)	**1.18 (1.00–1.38)**	1.19 (0.99–1.44)	1.12 (0.86–1.48)
Interaction between education and wave						
Low versus high education						
Wave 2 vs. wave 1	0.80 (0.53–1.22)	0.94 (0.54–1.63)	0.64 (0.33–1.22)	0.89 (0.56–1.41)	1.22 (0.70–2.14)	0.53 (0.25–1.12)
Wave 3 vs. wave 1	1.07 (0.70–1.77)	0.98 (0.55–1.75)	1.18 (0.61–2.30)	0.79 (0.48–1.29)	1.12 (0.63–2.00)	0.45 (0.20–1.05)
Wave 4 vs. wave 1	0.69 (0.43–1.11)	0.77 (0.41–1.43)	0.58 (0.28–1.20)	0.62 (0.36–1.05)	0.71 (0.37–1.36)	0.54 (0.23–1.26)
Wave 5 vs. wave 1	0.65 (0.41–1.04)	0.92 (0.50–1.70)	**0.41 (0.20–0.85)**	0.82 (0.48–1.38)	1.10 (0.59–2.05)	0.55 (0.23–1.34)
Intermediate versus high education						
Wave 2 vs. wave 1	0.79 (0.61–1.01)	0.98 (0.71–1.37)	**0.62 (0.42–0.90)**	0.93 (0.72–1.21)	1.03 (0.75–1.42)	0.82 (0.54–1.23)
Wave 3 vs. wave 1	0.91 (0.70–1.17)	0.99 (0.71–1.39)	0.84 (0.57–1.24)	0.94 (0.72–1.22)	1.07 (0.78–1.46)	0.82 (0.54–1.24)
Wave 4 vs. wave 1	0.79 (0.61–1.04)	1.10 (0.77–1.59)	**0.52 (0.35–0.77)**	**0.71 (0.54–0.94)**	0.85 (0.60–1.19)	**0.54 (0.35–0.84)**
Wave 5 vs. wave 1	**0.67 (0.52–0.87)**	0.89 (0.62–1.26)	**0.50 (0.34–0.75)**	1.12 (0.84–1.49)	1.38 (0.99–1.95)	0.80 (0.51–1.26)

^*^Bold indicates statistical significant (*P* < 0.05)

^a^Wave 1: November 2019, Wave 2: July 2020, Wave 3: November 2020, Wave 4: March 2021, Wave 5: November–December 2021

^b^Analyses were corrected for sex and age

^c^Analyses were corrected for age

^d^Time is presented for the reference group, the high educated workers

Among both men and women, low educated workers reported more often poor health compared to high educated workers. Significant interactions between time and educational level showed that educational inequalities in poor health gradually narrowed over time between intermediate and high educated men, and between low and high educated men (Table 2). No significant interactions between time and educational level were found for women.

**Fig. 2:**
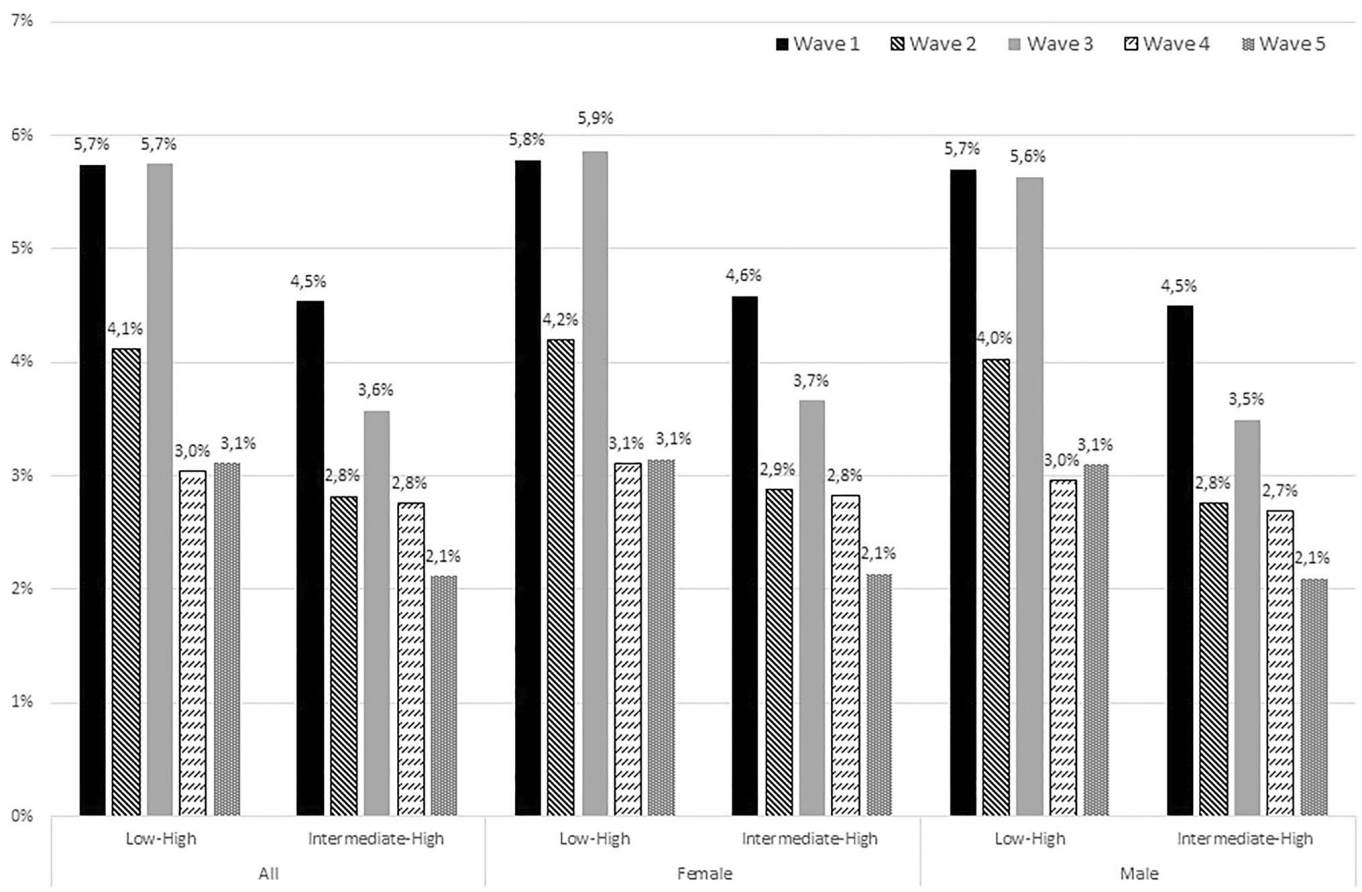
The absolute differences in poor self-rated health between educational groups for each wave

The absolute probabilities for poor health decreased in all educational groups until wave 4, whereafter the probabilities increased in the direction of the baseline value at Wave 5 (supplementary file 1). Figure 2 shows that the absolute differences in poor health followed the same trend as the relative educational inequalities, meaning that it narrowed between educational groups during the pandemic. For example, the absolute prevalence of poor health for low educated workers was 5.7% higher than for high educated workers at baseline. This difference decreased to 4.1% in Wave 2, increased to 5.7% in Wave 3, and decreased to 3.0% in Wave 4 and 3.1% in Wave 5 (Fig. 2). This trend was observed in both men and women (Fig. 2; supplementary file 2).

### Emotional exhaustion

Intermediate educated workers less often reported emotional exhaustion at baseline compared to high educated workers (OR: 0.71 (95% CI 0.56–0.90; Table 2)), while no statistically significant differences were found between low and high educated workers. Educational inequalities widened over time between high educated workers, and low and intermediate educated workers. However, this was only significant between intermediate and high educated workers between Wave 1 and Wave 4 (OR 0.71 (95% CI 0.54–0.94)). Between Wave 1 and Wave 5, there was no significant differences between educational groups in emotional exhaustion, indicating that educational inequalities narrowed between Wave 4 and Wave 5.

Results for men and women were slightly different. Intermediate educated women (OR 0.49; 95% CI 0.37–0.64) reported less emotional exhaustion than high educated women at baseline, while there was no difference for men. Relative educational inequalities in emotional exhaustion widened among men, and not among women.

**Fig. 3:**
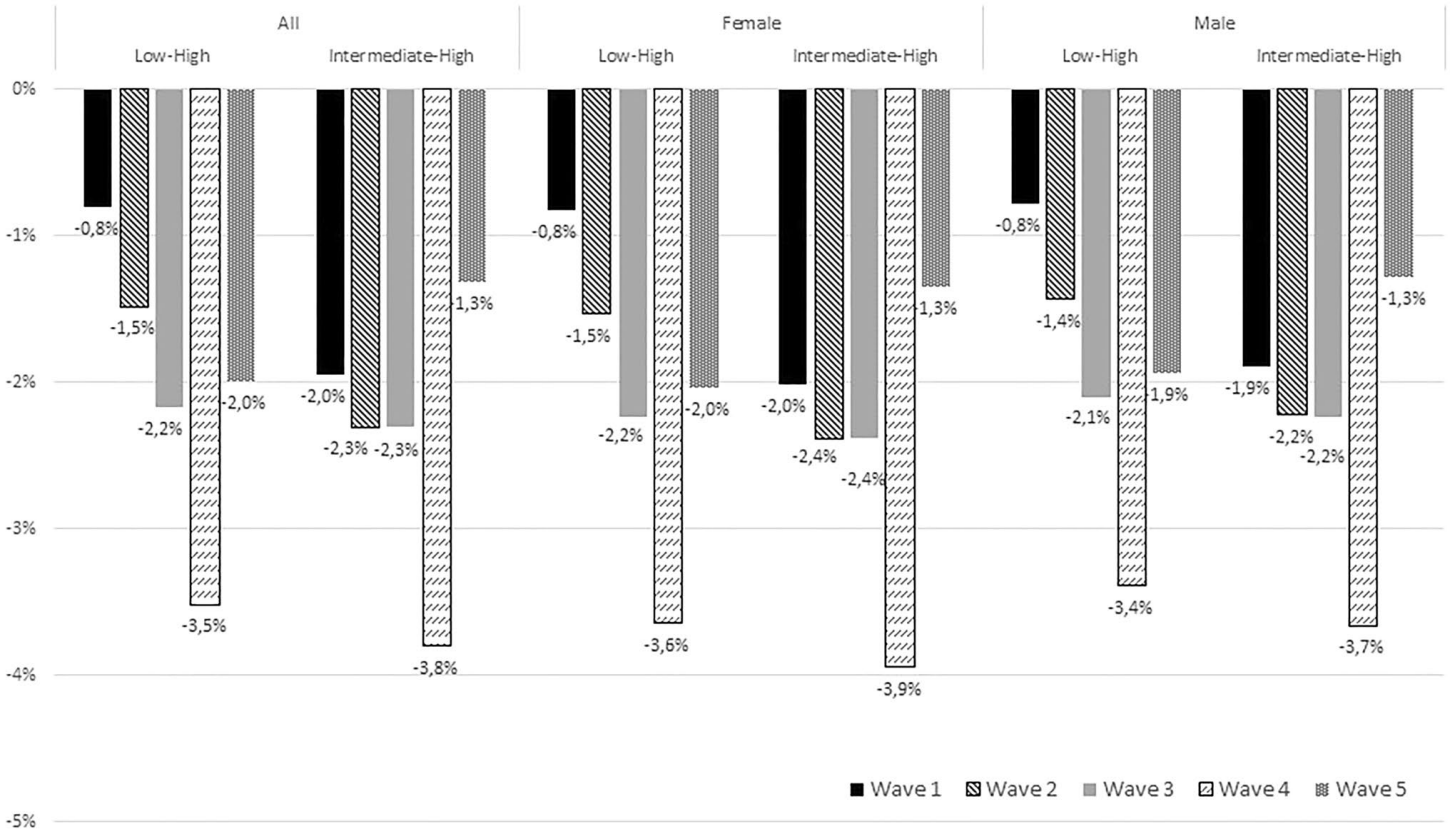
The absolute differences in emotional exhaustion (%) between educational groups for each wave

Over time, the absolute probabilities for emotional exhaustion slightly decreased for low and intermediate educated workers and was stable among high educated workers (Fig. 3; Supplementary file 2). As a result, absolute differences in the probability of emotional exhaustion between low and high educated workers as well as intermediate educated workers gradually increased between Wave 1 and 4, and decreased between Wave 4 and 5. The trends were similar for men and women.

## Discussion

Low and intermediate educated workers reported more often poor health than high educated workers across all waves. The prevalence of poor health first decreased across all educational levels between 2019 and the first year of the pandemic, and bounced back in December 2021. Relative educational inequalities in poor health reduced among men only until the last wave, and absolute differences slightly decreased among men and women. The prevalence of emotional exhaustion decreased over time among low and intermediate educated workers, but remained at the same level among high educated workers during the pandemic. Relative educational inequalities in emotional exhaustion widened among men up to Wave 4 and narrowed between the last two waves. The absolute differences in emotional exhaustion increased among both men and women in the first year of the pandemic, but narrowed between the last two waves.

The first methodological issue of the current study to address regards the non-response patterns over time, which might have influenced estimated trends in health and educational inequalities (Molenberg et al. 2021). In the current study, younger workers, low and intermediate educated workers, male workers and those with a poor health were less likely to respond in several waves (Supplementary file 3). Workers who were emotional exhausted at Wave 2 were less likely to respond in Wave 3. The decrease in poor health and emotional exhaustion between Wave 3 and Wave 4 may partly be explained by selective response during follow-up. It should be noticed that Wave 3 and Wave 4 took place within a period of increasing infections and hospitalizations and further restrictions in opening hours and closure of other sectors. The response among workers with high work demands might be lower in these waves, which might partly explain the decrease in emotional exhaustion. Second, we investigated if selective response differed across educational groups. In general, the patterns in non-response did not differ across educational groups (Supplementary file 3). Hence, it is unlikely that the non-response patterns influenced the trends in educational health inequalities.

The decrease of the prevalence of poor health among all workers during the first period of the pandemic was surprising, and in contrast with a study among a general population in the United States of America (Lee and Singh 2021). This U.S. study showed a slight increase in the proportion of people with poor health among all educational groups in the first period of the pandemic (Lee and Singh 2021). The decrease in the prevalence of poor health during the first year in the present study and the increase between the last two waves cannot only be explained by selective response, but also by other reasons. First, mitigation measures, including distancing and wearing masks, taken to reduce the number of COVID-19 infections also resulted in a decline of flu cases (Stamm et al. 2021) and occupational accidents (Statistics Netherlands 2022). This is especially true for the first year of the pandemic (Wave 2–Wave 4), which might reflect the lower prevalence of poor health during this period. Second, it could be hypothesized that individuals not directly affected by the most severe consequences of the pandemic (e.g., hospitalizations and deaths due to COVID-19, temporary closure of sectors) better value their own health. This is reflected in the relatively low prevalence of poor health in Wave 3 (November 2020) and Wave 4 (March 2021) of the current study. During these waves, the governmental measures were strict because of the increasing number of infected cases in the Netherlands. Even though the educational inequalities narrowed during the COVID-19 pandemic in the current study, we need to be aware that the prevalence of poor health is still the highest among low educated workers at all waves.

Regarding emotional exhaustion, only longitudinal studies that investigated the effects of the COVID-19 pandemic on different aspects of mental health (e.g., depression, anxiety) among the general working population were published. These studies showed no changes in educational differences in the prevalence of psychological distress during the first six months of the pandemic (Davillas and Jones 2021; Pierce et al. 2021). Gao et al. (2021) studied mental wellbeing of the general working population in the pre-pandemic situation up to 1 year into the pandemic. They showed that mental wellbeing decreased during strict lockdowns and COVID-19 peaks but also improved between these COVID-19 peaks. In the current study, mental health was operationalized as emotional exhaustion, which is defined as a state of feeling emotionally worn-out and drained as a result of accumulated stress and is one of the signs of a burn-out (Durand-Moreau 2019). The relatively high prevalence of emotional exhaustion of high educated workers may be attributed by the characteristics of this group. Half of these high educated workers worked in the education or health care sector. They played a crucial role in the societal response to the pandemic, which might have led to more work-related stress. Recent research already showed high burn-out rates among teachers (Nabe-Nielsen et al. 2021) and health care workers (Ghahramani et al. 2021) during the pandemic. However, following the recommendations of the government, the majority of the other high educated workers mainly worked from home, which might have resulted in less emotional exhaustion for some workers. Consequently, the prevalence of emotional exhaustion of high educated workers remained stable over time. We found a decreasing prevalence of emotional exhaustion among low and intermediate educated workers between Wave 2 and 4. During these waves, the governmental measures were the most stringent with closure or restrictions in opening hours of i.e., retail, sport clubs, restaurants and bars, resulting in less working activities. As mainly low and intermediate educated workers are working in these industries, this might explain the decrease in the prevalence of emotional exhaustion in these groups due to more time off and less work stress. However, these low and intermediate educated workers were more likely to face stress due to job insecurity and job loss compared to high educated workers. Previous studies showed that job insecurity and job loss are strongly related to depression and anxiety and to a lesser extent to emotional exhaustion (Rönnblad et al. 2019; Niedhammer et al. 2021). A recent review indeed showed higher levels of depression symptoms during the COVID-19 pandemic among those with a lower income (Parenteau et al. 2022).

The strength of the current study is the longitudinal dataset, which consisted of a large sample of workers including measurements before and during the COVID-19 pandemic. This enables not only studying the educational inequalities during the pandemic, but also to compare workers’ health with the period before the pandemic. The current study, however, is not without limitations. First, the NWCS-COVID-19 is a follow-up study of the annual NWCS study, and only workers who had granted permission in 2019 were invited to participate in the current study. Some additional analyses showed that mainly older and higher educated workers participated in the follow-up measurements compared to the original NWCS 2019 study. However, these analyses also showed that workers that participated in the follow-up study did not differ in self-rated health and emotional exhaustion compared to those that not participated (data not shown). This indicates that the investigated trends in health may not have been affected by selection at baseline. Additionally, half of the high educated workers were working in the health care or education sectors, which is higher than the one-third of the Dutch working population that is actually working in these sectors. This limits generalizability and should be taken into account when extrapolating the findings of the present study.

Third, the severity of the COVID-19 pandemic in terms of hospitalizations and governmental measures differed over time. These measures have probably led to early exits from work for some workers. However, workers who lost their work during follow-up were not included in the current study. Based on previous research on early exit from work to unemployment (van Rijn et al. 2014), it can be hypothesized that those workers that left paid employment were in worse health compared to those that remain in paid employment.

## Conclusion

This study confirmed that the prevalence of poor health is the highest among low educated workers. More strikingly, during the COVID-19 pandemic the existing educational inequalities in poor health narrowed in both relative terms for men and absolute terms for both men and women. Regarding emotional exhaustion, the prevalence decreased among low and intermediate workers in the first year of the pandemic, while a continuous higher prevalence of emotional exhaustion was observed among high educated workers. The educational inequalities for emotional exhaustion widened in relative terms for men and in absolute terms for men and women in the first one and a half year of the pandemic, but returned all back to pre-COVID-19 levels after two years. In summary, this study indicates that well-known educational inequalities in poor health and emotional exhaustion have not increased during the COVID-19 pandemic among Dutch workers.

## Supplementary Information

Below is the link to the electronic supplementary material.

Supplementary file1 (DOCX 44 KB)

## Data Availability

Data are stored at TNO, Unit Healthy Living in the Netherlands. Data are available upon reasonable request by the last author.
