# Supplementary material for: Educational inequalities in self-rated health and emotional exhaustion among workers during the COVID-19 pandemic: a longitudinal study
Source: Int Arch Occup Environ Health. 2022 Nov 2;96(3):401–10. doi: 10.1007/s00420-022-01931-y (PMC9628589; doi:10.1007/s00420-022-01931-y)
Supplement: Supplementary file 1 — Supplementary file1 (DOCX 44 KB) [file 420_2022_1931_MOESM1_ESM.docx]

**Supplementary file 1.**

**
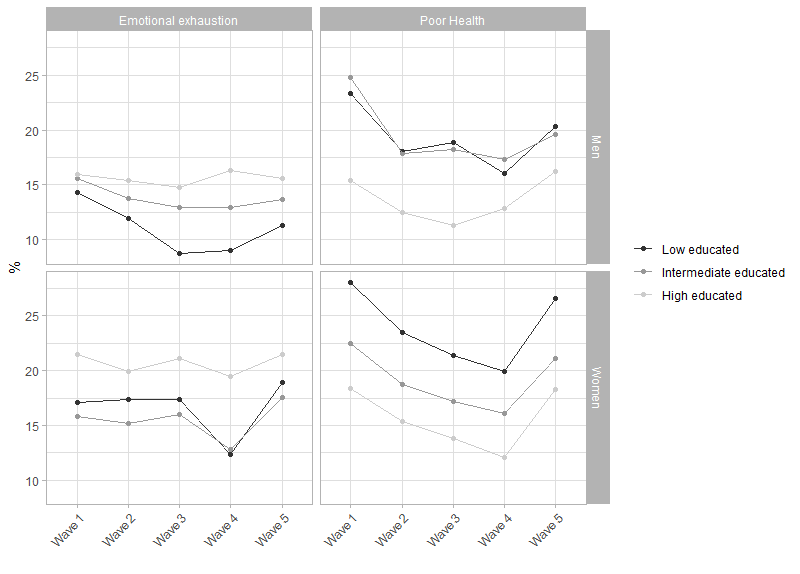
**

**Figure 1.**  Prevalence of poor health and emotional exhaustion for all educational levels, stratified by gender

**Supplementary file 2.** Predicted probabilities for poor health and burnout across educational groups, adjusted for age and gender (for all workers, and stratified by gender).

| **Poor health** | | | | | | | | | |
| --- | --- | --- | --- | --- | --- | --- | --- | --- | --- |
|  | **All** | | | **Female** | | | **Male** | | |
|  | Low | Intermediate | High | Low | Intermediate | High | Low | Intermediate | High |
| Wave 1 | 22% (1%-44%) | 21% (1%-39%) | 16% (0%-24%) | 23% (1%-47%) | 21% (1%-42%) | 17% (0%-26%) | 21% (1%-41%) | 20% (1%-36%) | 15% (0%-21%) |
| Wave 2 | 17% (0%-27%) | 16% (0%-23%) | 13% (0%-15%) | 18% (0%-30%) | 17% (0%-25%) | 14% (0%-17%) | 16% (0%-25%) | 15% (0%-21%) | 12% (0%-14%) |
| Wave 3 | 18% (0%-30%) | 16% (0%-23%) | 12% (0%-13%) | 19% (0%-32%) | 16% (0%-25%) | 13% (0%-15) | 17% (0%-27%) | 15% (0%-21%) | 11% (0%-12%) |
| Wave 4 | 15% (0%-21%) | 15% (0%-20%) | 12% (0%-13%) | 16% (0%-23%) | 15% (0%-22%) | 13% (0%-14) | 14% (0%-19%) | 14% (0%-18%) | 11% (0%-12%) |
| Wave 5 | 20% (1%-38%) | 19% (1%-34%) | 17% (0%-27%) | 21% (1%-41%) | 20% (1%-37%) | 18% (0%-29%) | 19% (1%-35%) | 18% (0%-32%) | 16% (0%-25%) |
|  |  |  |  |  |  |  |  |  |  |
| **Emotional exhaustion** | | | | | | | | | |
|  | **All** | | | **Female** | | | **Male** | | |
|  | Low | Intermediate | High | Low | Intermediate | High | Low | Intermediate | High |
| Wave 1 | 15% (0%-22%) | 14% (0%-19%) | 16% (0%-25%) | 17% (0%-26%) | 16% (0%-22%) | 18% (0%-29%) | 14% (0%-18%) | 13% (0%-15%) | 15% (0%-20%) |
| Wave 2 | 14% (0%-19%) | 14% (0%-17%) | 16% (0%-23%) | 16% (0%-23%) | 15% (0%-20%) | 17% (0%-28%) | 13% (0%-15%) | 12% (0%-14%) | 14% (0%-19%) |
| Wave 3 | 14% (0%-19%) | 14% (0%-19%) | 17% (0%-26%) | 16% (0%-23%) | 16% (0%-22%) | 18% (0%-30%) | 13% (0%-15%) | 13% (0%-15%) | 15% (0%-11%) |
| Wave 4 | 13% (0%-15%) | 13% (0%-15%) | 16% (0%-25%) | 14% (0%-18%) | 14% (0%-18%) | 18% (0%-29%) | 12% (0%-12%) | 11% (0%-12%) | 15% (0%-21%) |
| Wave 5 | 15% (0%-21%) | 16% (0%-23%) | 17% (0%-28%) | 17% (0%-25%) | 17% (0%-28%) | 19% (0%-32%) | 14% (0%-17%) | 14% (0%-19%) | 16% (0%-23%) |

**Supplementary file 3. Response over time**

**Table 3.1** Univariate analyses for personal characteristics and outcomes^1^ between non-responders and responders for each specific wave^2^

|  | **Wave 2** | **Wave 3** | **Wave 4** | **Wave 5** |
| --- | --- | --- | --- | --- |
|  | OR (95%CI) | OR (95%CI) | OR (95%CI) | OR (95%CI) |
| Age | **0.99 (0.99; 0.99)** | **0.99 (0.98; 0.99)** | **0.98 (0.98; 0.98)** | **0.98 (0.98; 0.99)** |
| Gender (male) | 0.98 (0.91; 1.06) | 1.14 (0.66; 1.22) | **1.13 (1.05; 1.21)** | 1.00 (0.93; 1.07) |
| Education |  |  |  |  |
| - Low | 1.00 (0.86; 1.15) | **1.25 (1.09; 1.44)** | **1.19 (1.04; 1.36)** | **1.31 (1.14; 1.49)** |
| - Intermediate | 1.04 (0.96; 1.13) | **1.11 (1.03; 1.20)** | 1.04 (0.97; 1.13) | **1.10 (1.02; 1.19)** |
| - High | Ref. | Ref. | Ref. | Ref. |
|  |  |  |  |  |
| Emotional exhaustion | 1.03 (0.93; 1.14) | **1.15 (1.05; 1.27)** | 1.07 (0.94; 1.22) | 0.96 (0.81; 1.14) |
| Poor health | 0.95 (0.86; 1.05) | **1.27 (1.15; 1.40)** | **1.18 (1.04; 1.35)** | 1.15 (0.98; 1.35) |

^1^Outcomes and characteristics were retrieved from the previous wave; ^2^Bold indicates significant differences (p<0.05)

**Table 3.2** Univariate analyses for personal characteristics and outcomes^1^ between low-educated non-responders and low-educated responders for each specific wave^2^

|  | **Wave 2** | **Wave 3** | **Wave 4** | **Wave 5** |
| --- | --- | --- | --- | --- |
|  | OR (95%CI) | OR (95%CI) | OR (95%CI) | OR (95%CI) |
| Age | 1.00 (0.99; 1.01) | **0.98 (0.97; 0.99)** | **0.98 (0.97; 0.99)** | **0.98 (0.97; 0.99)** |
| Gender (male) | 1.19 (0.91; 1.56) | 1.26 (0.97; 1.63) | **1.32 (1.02; 1.70)** | 1.19 (0.92; 1.53) |
|  |  |  |  |  |
| Emotional exhaustion | 0.72 (0.47; 1.09) | 1.23 (0.85; 1.79) | 1.11 (0.65; 1.92) | 1.10 (0.61; 1.98) |
| Poor health | 0.87 (0.62; 1.23) | **1.37 (1.00; 1.88)** | 1.03 (0.65; 1.62) | 1.34 (0.81; 2.23) |

^1^Outcomes and characteristics were retrieved from the previous wave; ^2^Bold indicates significant differences (p<0.05)

**Table 3.3** Univariate analyses for personal characteristics and outcomes^1^ between intermediate-educated non-responders and intermediate-educated responders for each specific wave^2^

|  | **Wave 2** | **Wave 3** | **Wave 4** | **Wave 5** |
| --- | --- | --- | --- | --- |
|  | OR (95%CI) | OR (95%CI) | OR (95%CI) | OR (95%CI) |
| Age | **0.98 (0.97; 0.99)** | **0.98 (0.98;0.99)** | **0.97 (0.97; 0.98)** | **0.98 (0.97; 0.98)** |
| Gender (male) | 1.19 (0.93; 1.53) | 1.10 (0.97; 1.25) | 1.10 (0.97; 1.24) | 0.95 (0.84; 1.07) |
|  |  |  |  |  |
| Emotional exhaustion | 1.09 (0.91; 1.30) | 1.27 (1.06; 1.51) | 1.14 (0.90; 1.44) | 1.04 (0.78; 1.42) |
| Poor health | 0.94 (0.80; 1.11) | **1.20 (1.03; 1.40)** | 1.16 (0.94; 1.43) | 1.00 (0.75; 1.32) |

^1^Outcomes and characteristics were retrieved from the previous wave; ^2^Bold indicates significant differences (p<0.05)

**Table 3.4** Univariate analyses for personal characteristics and outcomes^1^ between high-educated non-responders and high-educated responders for each specific wave^2^

|  | **Wave 2** | **Wave 3** | **Wave 4** | **Wave 5** |
| --- | --- | --- | --- | --- |
|  | OR (95%CI) | OR (95%CI) | OR (95%CI) | OR (95%CI) |
| Age | **0.99 (0.99; 1.00)** | **0.98 (0.98; 0.99)** | **0.99 (0.98; .099)** | **0.98 (0.98; 0.99)** |
| Gender (male) | 0.98 (0.89; 1.08) | **1.13 (1.02; 1.24)** | **1.12 (1.02; 1.23)** | 0.99 (0.91; 1.09) |
|  |  |  |  |  |
| Emotional exhaustion | 1.04 (0.92; 1.18) | 1.11 (0.98; 1.26) | 1.03 (0.88; 1.21) | 0.91 (0.73; 1.13) |
| Poor health | 0.96 (0.84; 1.10) | **1.27 (1.11; 1.46)** | **1.25 (1.05; 1.49)** | **1.20 (0.96; 1.50)** |

^1^Outcomes and characteristics were retrieved from the previous wave; ^2^Bold indicates significant differences (p<0.05)
